# Analysis by TeloView^®^ Technology Predicts the Response of Hodgkin’s Lymphoma to First-Line ABVD Therapy

**DOI:** 10.3390/cancers16162816

**Published:** 2024-08-10

**Authors:** Hans Knecht, Nathalie Johnson, Marc N. Bienz, Pierre Brousset, Lorenzo Memeo, Yulia Shifrin, Asieh Alikhah, Sherif F. Louis, Sabine Mai

**Affiliations:** 1 Division of Hematology, Jewish General Hospital, McGill University, Montréal, QC H3A 0G4, Canada; nathalie.johnson@mcgill.ca (N.J.); marc.bienz@gmail.com (M.N.B.); 2 Toulouse Cancer Center, Université de Toulouse, 31000 Toulouse, France; brousset.pierre@iuct-oncopole.fr; 3 Pathology Unit, Department of Experimental Oncology, Mediterranean Institute of Oncology, 95029 Viagrande, Italy; lorenzo.memeo@grupposamed.com; 4 Telo Genomics Corp., Toronto ON M5G 1L7, Canada; yulia.shifrin@telodx.com (Y.S.); asieh.alikhah@telodx.com (A.A.); sherif.louis@telodx.com (S.F.L.); 5 Department of Physiology and Pathophysiology, University of Manitoba, Winnipeg, MB R3T 2N, Canada; sabine.mai@umanitoba.ca; 6 CancerCare Manitoba Research Institute, CancerCare Manitoba, Winnipeg, MB R3E 0V9, Canada

**Keywords:** Hodgkin lymphoma, predictive model, relapse, ABVD therapy, prognostic biomarker, 3D telomere profiling

## Abstract

**Simple Summary:**

Three-dimensional (3D) telomere analysis using TeloView v 2.2 has shown promise in quantifying genomic instability and predicting therapy response. In a study of 156 Classic Hodgkin’s lymphoma (cHL), we identified significant 3D telomere parameters that predict patient outcomes. A predictive model using four 3D telomere parameters, including the proportion of t-stumps (very short telomeres), achieved an area under curve (AUC) of 0.83, with a sensitivity and specificity of 0.82 and 0.78, respectively.

**Abstract:**

Classic Hodgkin’s lymphoma (cHL) is a curable cancer with a disease-free survival rate of over 10 years. Over 80% of diagnosed patients respond favorably to first-line chemotherapy, but few biomarkers exist that can predict the 15–20% of patients who experience refractory or early relapsed disease. To date, the identification of patients who will not respond to first-line therapy based on disease staging and traditional clinical risk factor analysis is still not possible. Three-dimensional (3D) telomere analysis using the TeloView^®^ software platform has been shown to be a reliable tool to quantify genomic instability and to inform on disease progression and patients’ response to therapy in several cancers. It also demonstrated telomere dysfunction in cHL elucidating biological mechanisms related to disease progression. Here, we report 3D telomere analysis on a multicenter cohort of 156 cHL patients. We used the cohort data as a training data set and identified significant 3D telomere parameters suitable to predict individual patient outcomes at the point of diagnosis. Multivariate analysis using logistic regression procedures allowed for developing a predictive scoring model using four 3D telomere parameters as predictors, including the proportion of t-stumps (very short telomeres), which has been a prominent predictor for cHL patient outcome in a previously published study using TeloView^®^ analysis. The percentage of t-stumps was by far the most prominent predictor to identify refractory/relapsing (RR) cHL prior to initiation of adriamycin, bleomycin, vinblastine, and dacarbazine (ABVD) therapy. The model characteristics include an AUC of 0.83 in ROC analysis and a sensitivity and specificity of 0.82 and 0.78 respectively.

## 1. Introduction

The pathogenesis of classical Hodgkin’s Lymphoma (cHL) has been studied extensively over the past few decades [1,2,3]. The diagnostic tumor cell in cHL is the bi- or multinucleated Reed–Sternberg (RS) whereas the mononuclear Hodgkin (H) cell is the precursor of the RS cell [1,2]. Newly diagnosed patients are treated with combinations of chemotherapeutics, the most common of which include adriamycin, bleomycin, vinblastine, and dacarbazine (ABVD) [4,5,6]. Approximately 70% of newly diagnosed patients with stage III or IV advanced cHL show favorable outcomes in response to ABVD treatment resulting in long-lasting remission [7,8]. The remaining 30% of these high-risk cHL patients will either show primary resistance to ABVD or relapse during or after the completion of therapy and need salvage chemotherapy [9,10]. Even though cHL biology has been extensively investigated and well defined, a robust biomarker to predict initial anthracycline-based treatment failure remains an unresolved challenge in the management of the cHL disease.

Refractory and relapsing cHL patients can be treated with several other treatment modalities including autologous stem-cell transplantation (ASCT), brentuximab vedotin, and the PD-1 inhibitors nivolumab and pembrolizumab [11,12,13,14,15]. The improvement in the outcomes of refractory and relapsed cHL patients treated with such advanced therapeutics presented a rationale to advance such therapeutics to first-line therapy for all newly diagnosed cHL patients [16]. However, these therapeutics are accompanied by higher rates of neuropathy, neutropenia, and hypothyroidism compared to ABVD [14,17], and present a significantly higher cost burden on the healthcare systems as compared to traditional ABVD therapy [18,19]. Despite the development of these advanced therapies, the absence of a tool to stratify cHL patients at diagnosis remains a critical unmet clinical need yet to be achieved for cHL management.

Genomic instability is a hallmark of cancer and often plays a critical role in both cancer initiation and disease progression, influencing the overall prognosis of cancer patients [20,21]. Telomere dysfunction has been established to be one form of genomic instability that may trigger several other genomic instability events [22,23,24,25,26,27]. Furthermore, genomic instability and telomeric dysfunction have been associated with cHL [28,29], and have been described as the driving force that influences the degree of aggressiveness of the disease [30,31,32,33,34].

Three-dimensional (3D) telomeres analysis using TeloView^®^ technology has been shown to be a reliable tool for the assessment of genomic instability in several cancers including hematological disorders [35,36,37,38,39,40] and carcinomas [41,42,43,44]. TeloView^®^ analysis has been extensively used in a number of published studies addressing critical clinical questions regarding the biology of cHL [27,29,31,45,46,47].

Importantly, in a previous pilot study, we showed in a cohort including 32 cHL patients—16 long-lasting remissions versus 16 refractory early relapsing—that TeloView^®^ analysis prior to ABVD treatment, based on the level of telomere dysfunction, enables stratification of patients into a responder group and refractory/early relapse group [31].

To further test our hypothesis, that a high percentage of very short telomeres [25] (Xu and Blackburn) identified in diagnostic biopsies prior to ABVD treatment was associated with refractory or early relapsing disease (RR), we selected patients with either RR disease (within 12 months after completion ABVD therapy) or with for at least 5 years in complete remission (CR) after ABVD treatment and compared the telomere 3D profiles of both groups at time of diagnosis.

In this report, we validate the prognostic significance of telomere dysfunction using TeloView^®^ on a cohort of 156 cHL patients. Five telomere parameters measured by TeloView^®^ were highly associated with clinical outcome, with the most significant being the very high percentage of extremely short telomeres (t-stumps) present in H-cells of relapsed-refractory (RR) cases compared to H-cells of complete response (CR) cases. We further used the cohort as a training data set in multivariate regression analysis and identified telomere parameters suitable as predictors for modeling. With these data, we are able to report on prognostic predictive models that included telomere parameters with or without clinical risk factors, which can predict cHL evolution at the time of diagnosis with high specificity and sensitivity and at the individual patient level. We report an achieved AUC in ROC analysis of 0.83 with associated sensitivity and specificity of 0.82 and 0.78, respectively.

## 2. Materials and Methods

### 2.1. Study Design and Patient Information

This study was conducted retrospectively including 156 patients diagnosed with cHL. All patients included in the cohort were initially diagnosed with cHL by haemato-pathological assessment of lymph node biopsies and underwent ABVD chemotherapy as first-line treatment. Three independent hospitals/medical centers provided patient specimens and the corresponding de-identified clinical data. Each center obtained the appropriate jurisdictional ethics approval for this study. The participating hospitals/centers included: Jewish General Hospital—Montreal, Canada (69 cases); Mediterranean Institute of Oncology, Catania, Italy (64 cases); and Toulouse Cancer Center—Toulouse, France (23 cases). This study was conducted according to the Declaration of Helsinki. The cohort included 125 patients who went into remission for at least 5 years (identified as CR), and 31 patients who were either refractory to the treatment or relapsed within 12 months of diagnosis (identified as RR). All patients received ABVD as first-line therapy Table 1 summarizes the inclusion and exclusion criteria.

### 2.2. Tissue Specimens

This study was conducted blindly on sections of the initial diagnostic biopsy collected from each patient according to standard tissue collection and processing procedures. A total of 3 consecutive 5 μm tissue sections were obtained from the archived lymph node tissue biopsy of each patient. The biopsies were paraffin embedded according to guidelines of the College of American Pathologists (CAP) for immunohistochemistry (IHC) and fluorescence in situ hybridization (FISH) procedures. Hematoxylin-eosin (H&E) staining was performed on tissue section 1, CD30 IHC on tissue section 2, and quantitative 3D telomeres co-immuno-FISH on tissue section 3.

### 2.3. H&E and CD30 IHC

Consecutive tissue sections (slides) 1 and 2 were deparaffinized twice in xylene for 15 min each at room temperature then rehydrated in reverse-graded series of ethanol (100%, 75%, and 50% for 10 min each). The slides were then dipped briefly in ddH_2_O and then transferred into phosphate-buffered saline and used for H&E staining and anti-CD30 immunostaining. H&E and IHC staining were performed by Diagnostic Services Manitoba (Winnipeg, MB, Canada) according to standard clinical laboratory protocols), on a fee-for-service basis. These slides were then sent to the Telo Genomics Laboratory in Toronto to be analyzed (quality, best regions on the slide, sufficient number of H-and RS-cells) prior to quantitative 3D Telomere co-immuno-FISH Assay being performed on serial tissue section 3.

### 2.4. Quantitative 3D Telomere Co-Immuno-FISH Assay

Serial tissue section 3 of each patient was deparaffinized in two changes of Xylene, immersed into two changes of 100% ethanol and two changes of 95% ethanol for 3 min each. The ethanol treatment was followed by Antigen Retrieval (S169984-2, Agilent, Mississauga, ON, Canada) treatment for 30 min at 95 °C. For telomeres FISH hybridization, a Cy-3 labeled PNA telomere probe was applied (Agilent, Santa Clara, CA, USA). Denaturation was performed at 80 °C for 3 min followed by hybridization at 30 °C for two hours. The denaturation and hybridization were performed using a ThermoBright machine (Leica Biosystems, Wetzlar, IL, USA). After the hybridization, the slides were washed twice in washing buffer I (70% Formamide/10 mM Tris pH 7.5 ± 1) at room temperature for 15 min each, then in a washing buffer II 0.1X saline-sodium citrate (SSC, pH: 7.5 ± 1) for 5 min at 55 °C. To proceed with the anti-CD30 fluorescence immuno-staining, the slides were blocked in 4% BSA (Bovine Serum Albumin)/1X TBS blocking solution for 5 min at room temperature. The primary CD30 monoclonal mouse anti-Human antibody, clone Ber-H (M075129-2; DAKO, Singapore, Singapore) was used at a dilution of 1:20 followed by the secondary super-clonal goat-anti-mouse IgG (H+L) antibody conjugated to Alexa Fluor 488 (ThermoFisher, Norristown, PA, USA, cat# A28175) at a dilution of 1:100. Nuclei were counterstained with 4′6 Diamidino-2-phynylindole (DAPI) for visualization, followed by applying the antifade mounting medium Vectashield (Vector Laboratories, Burlington, ON, Canada).

### 2.5. 3D Image Acquisition and Processing

Images were acquired using a Zeiss Axio Imager Z.2 microscope (Zeiss, Jena, Germany) equipped with a Hamamatsu digital camera (Hamamatsu City, Japan, model# C11440-42U). Slides were first scanned with a 10x objective to identify areas rich in target cells based on the anti-CD30 staining. Three-dimensional images were then acquired using a 63x Zeiss immersion objective. Fifty-one focal planes spaced at 200 nm were captured along the *z*-axis (specimen depth) for every fluorochrome including Cy3 filter (telomeres), Spectrum green (CD30), and DAPI (nonspecific nuclear staining). The exposure time for Cy3 was 200 ms and Spectrum green 400 ms. Except for DAPI, the exposure time was locked for the entire cohort. The exposure for DAPI was assessed on a slide-by-slide basis. Images were then processed using a constrained iterative deconvolution algorithm [48], following which a single 3D image for each imaged field was constructed using the acquired 51 focal planes.

### 2.6. TeloView^®^ Analysis

Telomere parameters were quantified in 3D using the TeloView^®^ software platform [49,50]. (Telo Genomics, Toronto, ON, Canada). TeloView^®^ v 2.2 quantifies 6 primary molecular and structural telomeric parameters including 1—telomere length as a function of signal intensity; 2—number of telomere signals/nucleus; 3—number of telomeric aggregates (i.e., clusters of telomeres that are too close to be further resolved at an optical resolution limit of 200 nm); 4—nuclear volume; 5—*a*/*c* ratio (i.e., a spatial feature that assesses the cell cycle phase and is indicative of cell cycle progression); and 6—the distribution of telomeres relative to the nuclear periphery. Other relevant parameters may be derived from the 6 primary measured parameters including clustering telomeres based on size, average and total telomere length, average and total number of telomere signals/nucleus, and the percentage of telomeres stumps (very short telomeres with a relative fluorescent intensity from 0–5000 units).

***3D Image Analysis for Telomeres:*** Telomere measurements were conducted with TeloView^®^. By choosing a simple threshold for the telomeres, a binary image is found. Based on that, the center of gravity of intensities is calculated for every object resulting in a set of coordinates (*x*, y, z) denoted by crosses on the screen. The integrated intensity of each telomere is calculated because it is proportional to the telomere size [51].

(a) Nuclear volume: Nuclear volume within one 5 μm thin nuclear section of H-cells or RS-cells is calculated according to the 3D nuclear DAPI staining, as previously described [52]. Contrary to whole cell preparations (cells or cell lines), where the nuclei can be visualized with their entire volumes and *z*-stack analysis along the *z*-direction over 15 μm allows the calculation of the entire nuclear volume, in tissue sections the nuclear volume analysis is limited to 5 μm nuclear segments (as used as a standard for histopathologic diagnosis) along the z-direction. Deparaffinized tissue slides of 10 and 15 μm thickness are technically unsatisfactory for Q-FISH analysis. Thus, the nuclear volume represents about 30–50% of the total nuclear volume of H-cells (with a nuclear diameter of about 10–15 μm) and about 15–25% of the total nuclear volume of RS-cells (diameter of two up to several nuclei measures about 20–40 μm).
(b) Telomere number: The sum of all very small, small, mid-sized, and large telomeres and aggregates identified within one 5 μm thin segmental nuclear section of an H-cell or RS-cell.
(c) Telomere intensity: The sum of intensities of all very small, small, mid-sized, and large telomeres and aggregates identified within one 5 μm thin segmental nuclear section of an H-cell or RS-cell (viz. ∑ 2 × 15,000 units > ∑ 7 × 4000 units).
(d) Mean telomere intensity: Mean telomere relative fluorescent intensity (size) of all telomeres within a given segmental volume.
(e) Telomere size: Telomeres with a relative fluorescent intensity (*x*-axis) ranging from 0 to 5000 units are classified as very small (t-stumps), with an intensity ranging from 5000 to 15,000 units as small, with an intensity from 15,000 to 30,000 units as mid-sized, and with an intensity > 30,000 units as large [45].
(f) Telomere aggregates: Telomere aggregates are defined as clusters of telomeres that are found in close association and cannot be further resolved as separate entities at an optical resolution limit of 200 nm [53].

### 2.7. Statistical Analysis

General univariate *t*-test procedures were used to assess the relationship between the measured telomere parameters between the patient groups (CR versus RR) and to identify the parameters that are significantly different between the 2 groups and can be used as predictors. First, the telomeric parameters were assessed for equality of variances using Levene’s test. For the parameters with equal variance, Student’s *t*-test was performed, and for the parameters with unequal variance Welch’s *t*-test was performed.

The identified predictors were then verified for their suitability for modeling using confidence interval representation and NAPR1WAY analysis of variance. Multivariate logistic regression analysis was employed to generate predictive models using the identified suitable predictors. Receiver Operating Curves (ROC) were generated for the developed predictive models to assess the prediction power and to calculate the highest achievable positive and negative predictive values. The generated predictive models were assessed for confidence using the Likelihood Ratio, Wald, and Score tests.

## 3. Results

### 3.1. Cohort Clinical Data and Outcome

The 156 patients were evenly distributed across genders. 81% of patients were over the age of 50 years and 19% of patients were below the age of 50 years. Sixteen RR patients were under the age of 50 while 15 patients were over 50 years. A total of 125 patients remained in CR for at least 5 years after ABVD treatment, and 31 patients were primary refractory or relapsed within 12 months of diagnosis, ranging from 1 month to 11 months. All four disease clinical stages were represented in the cohort. The 31 relapsing patients included patients from all 4 clinical risk stages of cHL according to the initial assessment. For the 3D telomere analysis conducted in this study, only sections of the diagnostic biopsies were analyzed. A summary of the clinical data including patient demographics, clinical staging, and clinical outcomes is shown in Table 2.

### 3.2. Target cHL Tumor Cell Identification and Confirmation

**Figure 1:**
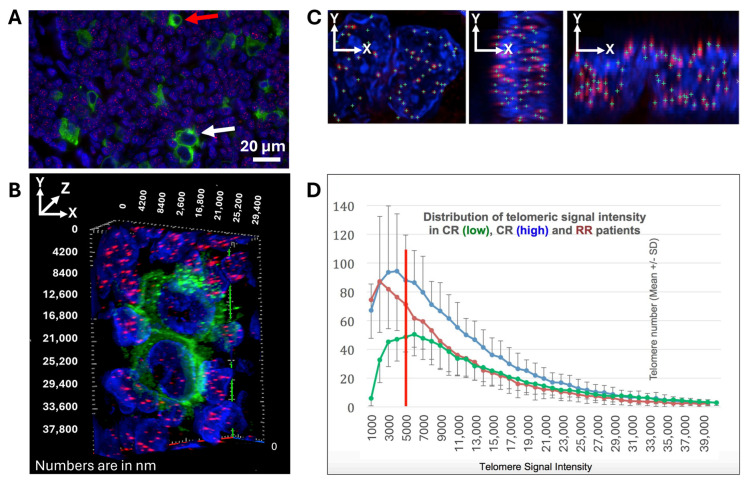
**Telomere co-immuno-FISH and TeloView^®^ analysis.** Telomeres are stained with a PNA probe tagged with a Cy3 fluorochrome (red); CD30 is stained with Alexa488 fluorochrome (green); nuclei are stained with the DNA stain DAPI (blue). (**A**) Representative 2D image showing H cells (red arrow) and RS cells (white arrow). (**B**) Representative 3D image showing binucleated RS cell shown in (**A**). Telomere loss and mainly small telomeres are easily recognizable when compared to surrounding reactive lymphocytes. (**C**) Representative illustration of telomere parameters quantification of RS cell shown in (**A**,**B**) using TeloView^®^ [50]. The telomere signals (green crosses) are marked in three axes (*x*, *y*, *z*). (**D**) Highly significant shift to t-stumps [23], extremely short telomeres, in RR (relapsing/refractory) cHL.

Cells were selected based on the positive immuno-staining with anti-CD30 antibody characteristic of H and RS cells (Figure 1A–C). Both mononucleated H cells and bi-multinucleated RS cells were included in the 3D telomere analysis. At least 30 H nuclei and 30 RS nuclei were analyzed per patient. CD-30-positive mononucleated cells were scored as H-cells and CD-30-positive bi-multinucleated cells were scored as RS cells. To confirm the accuracy of target cell selection, an internal second operator review was conducted. Only cells confirmed by two independent operators were considered for the analysis. Furthermore, the selected target cells of randomly chosen cases (34/156) were reviewed by a Hematopathologist. The concordance between the Operator cell selection and the Hematopathologist approval was >90%.

### 3.3. 3D Telomere Profiling of cHL Patients with RR Versus CR for Minimally 5 Years

The selected H and RS cells (30 H and 30 RS) for each patient were then analyzed using the software platform TeloView^®^ to quantify the six molecular and structural telomere parameters as described in the M&M section. *t*-test analysis was performed on grouped patient data points comparing all six measured TeloView^®^ parameters and the % of t-stumps for CR cHL patients versus RR cHL patients. When the *t*-test was applied to the mean of quantification of each parameter across the two patient groups (CR versus RR), statistical significance was achieved (*p* < 0.05; Table 3) for four out of the six primary telomere parameters, in addition to the most significant parameter, i.e., the % of t-stumps (telomeres with minimal signal intensity (length) shorter than 5000 a.u. (Figure 1D). The telomere dynamics of the mononuclear H-cells of the 31 cases with RR cHL (purple) are shown in red and plotted against the H-cells of the 31 CR cases (green) with the lowest and the 31 cases (blue) with the highest telomere numbers. The red bar at 5000 a.u. marks the border to the t-stumps at the left. RR cases culminate at a telomere length of 2000 a.u., whereas the CR cases with the lowest and highest telomere numbers culminate at 6000 a.u. and 4000 a.u., respectively. For both, the percentage of t-stumps is significantly lower compared to the RR group. The comparison of the 31 RR cases (purple) with the 31 CR cases with the highest telomere numbers (blue) reveals that despite a slightly elevated total number of the very short telomeres compared to the RR cases, their percentage of the total telomere number of the blue CR cases is significantly less as the percentage of the very short telomeres of the RR cases in relation to their total telomere number.

In brief, the average number of telomeres, average telomere length, average number of aggregates, and average nuclear volume achieved high significance, but not the A/C ratio or the distribution of the telomeres in nuclear space.

### 3.4. Regression Analysis and Predictive Modeling

We next performed confidence interval and NAPR1WAY analysis of variance to assess the relationship between the telomeric parameters and relapse status of patients, while accounting for cell-type (H or RS) and individual patient variability. The confidence interval and NAPR1WAY analyses identified four predictors suitable for regression analysis and predictive modeling. These predictors are the telomere length, nuclear volume, telomere aggregates, and t-stumps.

### 3.5. Predictive Modeling

**Figure 2:**
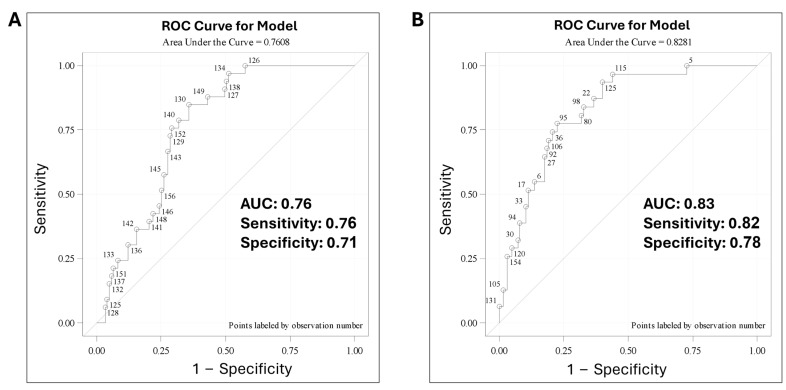
**Area Under the Curve (AUC), Sensitivity and Specificity.** (**A**) Predictive modeling including the four parameters telomere length, nuclear volume, telomere aggregates, and t-stumps. (**B**) Predictive modeling also integrating the clinical risk factors, namely age and disease stage.

Predictive modeling was performed using ROC curve analysis including different combinations of all the suitable predictors and their derivatives, to determine which combination of predictors will yield the highest Area Under the Curve (AUC), specificity, and sensitivity. Out of the generated models, the highest specificity and sensitivity were achieved using derivatives of all four predictors. The generated ROC curve had an AUC of 0.76, sensitivity of 0.76, and specificity of 0.71 (Figure 2A). We further investigated if integrating clinical risk factors namely age and disease stage, with the same telomere parameter, would impact the AUC, sensitivity, and specificity. For this purpose, we categorized patients based on age (<50 years or >50 years), and disease stage (stage I and IIA versus IIB, IIIA, IIIB, IVA, and IVB). The AUC of the generated ROC revealed a prediction power of 0.83 and achieved sensitivity and specificity of 0.82 and 0.78, respectively (Figure 2B). We examined the confidence of the generated ROCs using the Likelihood ratio, Wald, and Scoring tests. All models showed high confidence with *p*-values *p* < 0.001 (Table 4).

## 4. Discussion

Previous reports have demonstrated the utility of TeloView^®^ technology in assessing the correlation between cHL outcomes and telomere dysfunction [31,45,46]. Comparing patient groups, it was shown that the 3D analysis of telomeres using TeloView^®^ technology, conducted on a cohort of 32 HL patients, revealed distinct telomere profiles for cHL patients who were refractory or relapsed versus patients who remained in CR [31]. More recent reports characterized several molecular mechanisms showing an association between a dysfunctional Sheltrin complex [27,46] and Lamin A/C [47], with disease progression and transition of H cells to the end-stage RS cells.

Here, building on the strong scientific evidence demonstrated in the previous reports, we designed a retrospective clinical study to develop a predictive model for identifying patients with an aggressive form of the disease, who are at higher risk of refractory disease or relapse within one year from point of diagnosis, while being treated with ABVD.

cHL is categorized as a rare disease, consequently conducting such modeling studies prospectively is challenging given the difficulty of recruiting patients in longitudinal studies with years of follow-up. This study was conducted retrospectively with minimally 5 years of clinical follow-up. The study included a relatively adequate cohort size (156 Patients) for a rare disease. Furthermore, the cohort included sufficient representation of relapsing patients (20%) to allow for modeling analysis. The low level of events in cohorts is another challenge for developing reliable prognostic scoring models for rare diseases [54].

In concordance with previous reports [31,46], TeloView^®^ quantification revealed that RS cells across both patient groups showed higher numbers of detected telomere signals, nuclear volume, number of telomere aggregates, A/C ratio, telomere distribution space inside the nucleus and higher percentage of t-stumps when compared to H-cells. Meanwhile, RS cells of both patient groups (CR and RR) showed significantly decreased average telomere length as compared to the H cells of either patient group. The most significant differences were observed in the percentage of t-stumps between the H-cells of RR and CR cases (*p* = 0.000011) and RS-cells of RR and CR cases (*p* = 0.000077). This is related to the fact that the nuclear volume within one 5 μm thin nuclear section of H-cells or RS-cells is calculated according to the 3D nuclear DAPI staining, as previously described [49]. Contrary to whole cell preparations (cells or cell lines), where the nuclei can be visualized with their entire volumes and where *z*-stack analysis along the *z*-direction over 15 μm allows the calculation of the entire nuclear volume, in tissue sections the nuclear volume analysis is limited to 5 μm nuclear segments (as used as a standard for histopathologic diagnosis) along the z-direction. Deparaffinized tissue slides of 10 and 15 μm thickness are technically unsatisfactory for Q-FISH analysis. Thus, the nuclear volume represents a segmental nuclear volume including 30–50% of the total nuclear volume of H-cells (nuclear diameter of about 10–15 μm) and 15–25% of the total nuclear volume of RS-cells (diameter of two up to several nuclei about 20–40 μm). This may affect the total number of telomeres and aggregates depending on the cut level through an H- or RS-cell but will not influence the percentage of telomere length distribution (i.e., in a cross-section with less volume and consequently fewer telomeres, the percentage of very short telomeres remains the same). This distribution of data points across RS versus H cells is typical to the cHL disease biology, the multinucleated nature of the RS cells characteristic of the disease, and the ghost-cell phenomena commonly observed in the RS cells when examined using 3D telomere analysis.

In this context, it may be interesting to mention that even in Burkitt’s lymphoma with relatively monomorphic spherical malignant cells, size variation of the nuclei has been described in cases from different origins [55]. The situation is different from Richter’s transformation, a progression of CLL, a relatively benign disease, into a high-grade lymphoma type DLBCL, associated with a significant increase in the nuclear volume [56]. The length of telomeres is related to cell division cycles [57,58], or, in other words, the more divisions, the shorter the telomeres [59,60]. Tumor cells of highly aggressive lymphomas and other aggressive cancers divide more and have a short doubling time, short telomere, and high proliferative index, as shown by the high percentage of Ki-67^+^ cells [61,62].

The initial *t*-test for assessing the relationship between the patient groups was conducted on the mean of the data points of combined H and RS cells for each patient group. This approach yielded significant *p*-values in five out of the seven measured parameters. In previous reports, data points of H cells or RS cells were compared between patient groups. In this report, however, we intended to capture the significance of combining data points of both cell types for each patient group. Most significant was the difference in the number of t-stumps in remission cases versus refractory/relapsing cases at the point of diagnosis (*p* = 0.000011), confirming our initial observation is a small series [31].

Based on the initial *t*-test results, suitable predictor parameters were identified. We further vetted these parameters using confidence interval and NAPR1WAY analysis of variance to confirm the suitability of the parameters for regression modeling where individual patient variability will be accounted for. For this reason, we also examined derived parameters including quartile and ratio subgrouping of the predictors.

The generated ROCs achieved the highest AUC of 0.83 and the highest sensitivity and specificity of 0.82 and 0.78, respectively. This was achieved by integrating the clinical risk factors, age, and disease stage with the TeloView^®^ parameters. This integration achieved an improvement of approximately 7% over the model using TeloView^®^ parameters alone. 

Over the past two decades, the clinical and biological prognostic factors have substantially improved to predict the clinical course of cHL and to generate new treatment modalities. The most important step was the introduction of the international prognostic score by the German Hodgkin’s Lymphoma Study Group (GHSG) [63,64]. Further important prognostic factors are bulky disease and extra-lymphatic localizations [65] and tumor burden-defined PET-CT [66,67]. Other important prognostic biomarkers are beta2-microglobulin [68], circulating tumor DNA [69,70], and TARC [71]. The progress of risk stratification, management, and therapeutic progress of cHL has recently been reviewed [72] but there is still a gray zone in the identification of early-stage disease with aggressive behavior. New biomarkers might further reduce this percentage of RR patients and help to choose optimal upfront therapy. In this setting, it might be worth testing the 3D technology in a prospective study of high-risk patients treated with a newer therapeutic regimen.

In this study, patients were included based on uniform treatment with ABVD as first-line treatment. The rationale is ABVD was the most common first-line treatment of cHL in North America, effective on over 80% of newly diagnosed patients. Now, as treatment regimens evolved to include new agents or different regimens such as BEACOPP, we speculate that the assay will maintain its characteristics of specificity and sensitivity in the fact that we measure the level of genomic instability at diagnosis. A future validation will be beneficial.

In clinical practices, generally, it is preferred to resort to prognostic markers that will achieve >90 positive/negative predictive values, specificity, and sensitivity. However, there are numerous traditional and newly developed prognostic markers that are extensively used in the clinic for different indications with test characteristics below 60–70% [73]. These tests were accepted for the fact that a better alternative is not available. In this study, we present a sensitivity of >80 and specificity of over 75% generated from a 156-patient cohort to address a clinically unmet need, where no other tool is available to guide the clinical decision-making process.

## 5. Conclusions

The results of this study offer a long-awaited path to a precision medicine approach for the treatment of cHL patients. The ready-to-use predictive model will further be validated retrospectively on an independent patient cohort as a further step narrowing the gap to introduce the test to the clinic. The test can further be validated prospectively in longitudinal studies once the test is adopted in the clinic for health economy and utility validation.

## Figures and Tables

**Table 1:** Study inclusion and exclusion criteria.

Inclusion Criteria	Exclusion Criteria
First time diagnosed with cHL ^a^	Age < 18 and >80 years old
Availability of lymph node diagnostic tissue	
Availability of demographic and clinical follow up data	
First line treatment with ABVD ^b^	

^a^ classic Hodgkin’s Lymphoma. ^b^ adriamycin, bleomycin, vinblastine, and dacarbazine.

**Table 2:** Summary of the demographic and clinical data of the 156 multicenter cohort included in this study.

	Gender		Age Groups		Disease Stage (Costwold)				Response to ABVD	
	Male	Female	≥50 Years	<50 Years	I	II	III	IV	Remission ≥5 Years	Refractory or Relapse within 12 Months
*n* = 156	79	77	126	30	23	70	32	31	125	31

**Table 3:** The TeloView^®^ quantification of the 6 primary telomere parameters for H and RS cells of the cohort patients grouped by their clinical outcome.

	A—Remission > 5 Years (*n*-125)		B—Refractory/Relapse ≤ 12 Month (*n* = 31)		<HA + RSA> vs. <HB + RSB>	HA vs. HB	RSA vs. RSB
Telomere Parameter	HA	RSA	HB	RSB	*p*-Value	*p*-Value	*p*-Value
Number of telomeres	36 (17.8; 0.29)	55 (36; 0.6)	31 (14, 0.48)	47 (31; 1.1)	0.04 *	0.000014 *	0.0007 *
Number of Aggregates	4 (2.9; 0.05)	7 (5.5; 0.09)	3 (2.5; 0.08)	6 (4.8; 0.16)	0.034 *	0.0026 *	0.03 *
Average telomere length	2926 (1185; 39.5)	2599 (924; 15.4)	2788 (1132; 18.9)	2457 (871; 29)	0.0029 *	0.012 *	0.0003 *
Nuclear Volume (μ^3^)	469.31 (400.81.8; 6.68)	718.10 (686.11; 11.43)	440.08 (330.29; 11.00)	702.00 (574.54; 19.15)	0.017 *	0.4	0.5
A/C Ratio	3 (1.2; 0.02)	3.6 (1.5; 0.026)	2.8 (1.1; 0.04)	3.4 (1.4; 0.049)	0.078	0.42	0.08
Distribution of telomeres	3813 (777; 25.8)	4876 (1450.7; 24)	3684 (775; 25.8)	4740 (1428.4; 47.6)	0.085	0.00016 *	0.1
% of t-stumps	42 (14; 0.21)	57 (21, 0.33)	49 (17; 0.27)	84 (27; 39)	0.0012 *	0.000011 *	0.000077 *

A = Remission, B = Relapse/Refractory. The % of very short telomeres (t-stumps) representing telomere signals with fluorescence intensity < 5000 a.u is shown at the bottom of the table. The standard deviation and the standard error respectively are included between brackets after each value. The right of the table shows the *t*-test calculated *p*-values of the telomere parameters assessing the relationship between patient groups. The average number of telomeres (signals), average number of aggregates, average telomere length (signal intensity), and nuclear volume showed unequal variances and were assessed using Welch’s *t*-test. Average A/C ratio, average distribution of telomeres within the nucleus, and the % of t-stumps showed equal variance and were assessed using Student’s *t*-test. Significance was set at ≤0.05 and highlighted by the * symbol beside or below the *p*-value. HA and RSA pertain to the H&RS cells of patients who stayed in remission for >5 years, and HB and RSB pertain to the H and RS cells of patients who were refractory or relapsed within 12 months. *p*-values comparing the sum of H and RS cells of patient groups, and *p*-values comparing cell types across patient groups are both shown in the table.

**Table 4:** Confidence assessment of ROCs developed using TeloView^®^ parameters alone or combined with clinical risk factors.

Confidence Test	TeloView Parameters Only	TeloView with Clinical Risk Factors
Likelihood Ratio	0.0003	<0.0001
Score	0.0009	0.0003
Wald	0.0024	0.0011

## Data Availability

Data will be made available upon request from the corresponding author.

## References

[1] Piris M.A., Medeiros L.J., Chang K.-C. (2020). Hodgkin lymphoma: A review of pathological features and recent advances in pathogenesis. Pathology.

[2] Bienz M., Ramdani S., Knecht H. (2020). Molecular pathogenesis of Hodgkin lymphoma: Past, present, future. Int. J. Mol. Sci..

[3] Weniger M.A., Küppers R. (2021). Molecular biology of Hodgkin lymphoma. Leukemia.

[4] Canellos G.P., Anderson J.R., Propert K.J., Nissen N., Cooper M.R., Henderson E.S., Green M.R., Gottlieb A., Peterson B.A. (1992). Chemotherapy of advanced Hodgkin’s disease with MOPP, ABVD, or MOPP alternating with ABVD. N. Engl. J. Med..

[5] Kuruvilla J. (2009). Standard therapy of advanced Hodgkin lymphoma. ASH Educ. Program Book.

[6] Gordon L.I., Hong F., Fisher R.I., Bartlett N.L., Connors J.M., Gascoyne R.D., Wagner H., Stiff P.J., Cheson B.D., Gospodarowicz M. (2013). Randomized phase III trial of ABVD versus Stanford V with or without radiation therapy in locally extensive and advanced-stage Hodgkin lymphoma: An intergroup study coordinated by the Eastern Cooperative Oncology Group (E2496). J. Clin. Oncol..

[7] Merli F., Luminari S., Gobbi P.G., Cascavilla N., Mammi C., Ilariucci F., Stelitano C., Musso M., Baldini L., Galimberti S. (2016). Long-term results of the HD2000 trial comparing ABVD versus BEACOPP versus COPP-EBV-CAD in untreated patients with advanced Hodgkin lymphoma: A study by Fondazione Italiana Linfomi. J. Clin. Oncol..

[8] Carde P., Karrasch M., Fortpied C., Brice P., Khaled H., Casasnovas O., Caillot D., Gaillard I., Bologna S., Ferme C. (2016). Eight cycles of ABVD versus four cycles of BEACOPPescalated plus four cycles of BEACOPPbaseline in stage III to IV, international prognostic score ≥ 3, high-risk Hodgkin lymphoma: First results of the phase III EORTC 20012 Intergroup Trial. J. Clin. Oncol..

[9] Viviani S., Zinzani P.L., Rambaldi A., Brusamolino E., Levis A., Bonfante V., Vitolo U., Pulsoni A., Liberati A.M., Specchia G. (2011). ABVD versus BEACOPP for Hodgkin’s lymphoma when high-dose salvage is planned. N. Engl. J. Med..

[10] Johnson P.W. (2016). Response-adapted frontline therapy for Hodgkin lymphoma: Are we there yet?. Hematol. 2014 Am. Soc. Hematol. Educ. Program Book.

[11] Younes A., Gopal A.K., Smith S.E., Ansell S.M., Rosenblatt J.D., Savage K.J., Ramchandren R., Bartlett N.L., Cheson B.D., De Vos S. (2012). Results of a pivotal phase II study of brentuximab vedotin for patients with relapsed or refractory Hodgkin’s lymphoma. J. Clin. Oncol..

[12] Gopal A.K., Chen R., Smith S.E., Ansell S.M., Rosenblatt J.D., Savage K.J., Connors J.M., Engert A., Larsen E.K., Chi X. (2015). Durable remissions in a pivotal phase 2 study of brentuximab vedotin in relapsed or refractory Hodgkin lymphoma. Blood J. Am. Soc. Hematol..

[13] Ansell S.M., Lesokhin A.M., Borrello I., Halwani A., Scott E.C., Gutierrez M., Schuster S.J., Millenson M.M., Cattry D., Freeman G.J. (2015). PD-1 blockade with nivolumab in relapsed or refractory Hodgkin’s lymphoma. N. Engl. J. Med..

[14] Chen R., Zinzani P.L., Fanale M.A., Armand P., Johnson N.A., Brice P., Radford J., Ribrag V., Molin D., Vassilakopoulos T.P. (2017). Phase II study of the efficacy and safety of pembrolizumab for relapsed/refractory classic Hodgkin lymphoma. J. Clin. Oncol..

[15] Kuruvilla J., Ramchandren R., Santoro A., Paszkiewicz-Kozik E., Gasiorowski R., Johnson N.A., Fogliatto L.M., Goncalves I., de Oliveira J.S.R., Buccheri V. (2021). Pembrolizumab versus brentuximab vedotin in relapsed or refractory classical Hodgkin lymphoma (KEYNOTE-204): An interim analysis of a multicentre, randomised, open-label, phase 3 study. Lancet Oncol..

[16] Ansell S.M., Radford J., Connors J.M., Długosz-Danecka M., Kim W.-S., Gallamini A., Ramchandren R., Friedberg J.W., Advani R., Hutchings M. (2022). Overall survival with brentuximab vedotin in stage III or IV Hodgkin’s lymphoma. N. Engl. J. Med..

[17] Evens A.M., Connors J.M., Younes A., Ansell S.M., Kim W.S., Radford J., Feldman T., Tuscano J., Savage K.J., Oki Y. (2022). Older patients (aged ≥60 years) with previously untreated advanced-stage classical Hodgkin lymphoma: A detailed analysis from the phase III ECHELON-1 study. Haematologica.

[18] Huntington S.F., von Keudell G., Davidoff A.J., Gross C.P., Prasad S.A. (2018). Cost-effectiveness analysis of brentuximab vedotin with chemotherapy in newly diagnosed stage III and IV Hodgkin lymphoma. J. Clin. Oncol..

[19] Raymakers A., Costa S., Cameron D., Regier D.A. (2020). Cost-effectiveness of brentuximab vedotin in advanced stage Hodgkin’s lymphoma: A probabilistic analysis. BMC Cancer.

[20] Coleman W.B., Tsongalis G.J. (1995). Multiple mechanisms account for genomic instability and molecular mutation in neoplastic transformation. Clin. Chem..

[21] Pihan G.A., Purohit A., Wallace J., Knecht H., Woda B., Quesenberry P., Doxsey S.J. (1998). Centrosome defects and genetic instability in malignant tumors. Cancer Res..

[22] Blackburn E.H. (2001). Switching and signaling at the telomere. Cell.

[23] Maser R.S., DePinho R.A. (2002). Connecting chromosomes, crisis, and cancer. Science.

[24] De Lange T. (2005). Telomere-Related Genome Instability in Cancer.

[25] Xu L., Blackburn E.H. (2007). Human cancer cells harbor T-stumps, a distinct class of extremely short telomeres. Mol. Cell.

[26] Mai S. (2010). Initiation of telomere-mediated chromosomal rearrangements in cancer. J. Cell. Biochem..

[27] Lajoie V., Lemieux B., Sawan B., Lichtensztejn D., Lichtensztejn Z., Wellinger R., Mai S., Knecht H. (2015). LMP1 mediates multinuclearity through downregulation of shelterin proteins and formation of telomeric aggregates. Blood J. Am. Soc. Hematol..

[28] Knecht H., Sawan B., Lichtensztejn D., Lemieux B., Wellinger R., Mai S. (2009). The 3D nuclear organization of telomeres marks the transition from Hodgkin to Reed–Sternberg cells. Leukemia.

[29] Guffei A., Sarkar R., Klewes L., Righolt C., Knecht H., Mai S. (2010). Dynamic chromosomal rearrangements in Hodgkin’s lymphoma are due to ongoing three-dimensional nuclear remodeling and breakage-bridge-fusion cycles. Haematologica.

[30] Re D., Zander T., Diehl V., Wolf J. (2002). Genetic instability in Hodgkin’s lymphoma. Ann. Oncol..

[31] Knecht H., Kongruttanachok N., Sawan B., Brossard J., Prévost S., Turcotte E., Lichtensztejn Z., Lichtensztejn D., Mai S. (2012). Three-dimensional telomere signatures of Hodgkin-and Reed-Sternberg cells at diagnosis identify patients with poor response to conventional chemotherapy. Transl. Oncol..

[32] Knecht H., Righolt C., Mai S. (2013). Genomic instability: The driving force behind refractory/relapsing Hodgkin’s lymphoma. Cancers.

[33] Roemer M.G., Advani R.H., Ligon A.H., Natkunam Y., Redd R.A., Homer H., Connelly C.F., Sun H.H., Daadi S.E., Freeman G.J. (2016). PD-L1 and PD-L2 genetic alterations define classical Hodgkin lymphoma and predict outcome. J. Clin. Oncol..

[34] Cuceu C., Hempel W.M., Sabatier L., Bosq J., Carde P., M’kacher R. (2018). Chromosomal instability in Hodgkin lymphoma: An in-depth review and perspectives. Cancers.

[35] Gadji M., Fortin D., Tsanaclis A.-M., Garini Y., Katzir N., Wienburg Y., Yan J., Klewes L., Klonisch T., Drouin R. (2010). Three-dimensional nuclear telomere architecture is associated with differential time to progression and overall survival in glioblastoma patients. Neoplasia.

[36] Gadji M., Vallente R., Klewes L., Righolt C., Wark L., Kongruttanachok N., Knecht H., Mai S. (2011). Nuclear remodeling as a mechanism for genomic instability in cancer. Adv. Cancer Res..

[37] Gadji M., Adebayo Awe J., Rodrigues P., Kumar R., Houston D.S., Klewes L., Dièye T.N., Rego E.M., Passetto R.F., de Oliveira F.M. (2012). Profiling three-dimensional nuclear telomeric architecture of myelodysplastic syndromes and acute myeloid leukemia defines patient subgroups. Clin. Cancer Res..

[38] Klewes L., Vallente R., Dupas E., Brand C., Grün D., Guffei A., Sathitruangsak C., Awe J.A., Kuzyk A., Lichtensztejn D. (2013). Three-dimensional nuclear telomere organization in multiple myeloma. Transl. Oncol..

[39] Rangel-Pozzo A., Yu P.L.I., LaL S., Asbaghi Y., Sisdelli L., Tammur P., Tamm A., Punab M., Klewes L., Louis S. (2021). Telomere architecture correlates with aggressiveness in multiple myeloma. Cancers.

[40] Bienz M.N., Petrogiannis-Haliotis T., Pehr K., Benlimame N., Mai S., Knecht H. (2021). Three-Dimensional Telomeric Fingerprint of Mycosis Fungoides and/or Sézary Syndrome: A Pilot Study. J. Investig. Dermatol..

[41] Wark L., Danescu A., Natarajan S., Zhu X., Cheng S.-Y., Hombach-Klonisch S., Mai S., Klonisch T. (2014). Three-dimensional telomere dynamics in follicular thyroid cancer. Thyroid.

[42] Caria P., Dettori T., Frau D.V., Lichtenzstejn D., Pani F., Vanni R., Mai S. (2019). Characterizing the three-dimensional organization of telomeres in papillary thyroid carcinoma cells. J. Cell. Physiol..

[43] Drachenberg D., Awe J.A., Rangel Pozzo A., Saranchuk J., Mai S. (2019). Advancing risk assessment of intermediate risk prostate cancer patients. Cancers.

[44] Rangel-Pozzo A., Sisdelli L., Cordioli M.I.V., Vaisman F., Caria P., Mai S., Cerutti J.M. (2020). Genetic landscape of papillary thyroid carcinoma and nuclear architecture: An overview comparing pediatric and adult populations. Cancers.

[45] Knecht H., Sawan B., Lichtensztejn Z., Lichtensztejn D., Mai S. (2010). 3D Telomere FISH defines LMP1-expressing Reed–Sternberg cells as end-stage cells with telomere-poor ‘ghost’ nuclei and very short telomeres. Lab. Investig..

[46] Knecht H., Johnson N.A., Haliotis T., Lichtensztejn D., Mai S. (2017). Disruption of direct 3D telomere–TRF2 interaction through two molecularly disparate mechanisms is a hallmark of primary Hodgkin and Reed–Sternberg cells. Lab. Investig..

[47] Contu F., Rangel-Pozzo A., Trokajlo P., Wark L., Klewes L., Johnson N.A., Petrogiannis-Haliotis T., Gartner J.G., Garini Y., Vanni R. (2018). Distinct 3D structural patterns of lamin A/C expression in Hodgkin and Reed-Sternberg cells. Cancers.

[48] Schaefer L., Schuster D., Herz H. (2001). Generalized approach for accelerated maximum likelihood based image restoration applied to three-dimensional fluorescence microscopy. J. Microsc..

[49] Chuang T.C.Y., Moshir S., Garini Y., Chuang A.Y.-C., Young I.T., Vermolen B., Doel R.v.D., Mougey V., Perrin M., Braun M. (2004). The three-dimensional organization of telomeres in the nucleus of mammalian cells. BMC Biol..

[50] Vermolen B., Garini Y., Mai S., Mougey V., Fest T., Chuang T.C., Chuang A.Y., Wark L., Young I.T. (2005). Characterizing the three-dimensional organization of telomeres. Cytom. Part A J. Int. Soc. Anal. Cytol..

[51] Poon S.S., Martens U.M., Ward R.K., Lansdorp P.M. (1999). Telomere length measurements using digital fluorescence microscopy. Cytom. J. Int. Soc. Anal. Cytol..

[52] Mai S., Garini Y. (2006). The significance of telomeric aggregates in the interphase nuclei of tumor cells. J. Cell. Biochem..

[53] Sarkar R., Guffei A., Vermolen B.J., Garini Y., Mai S. (2007). Alterations of centromere positions in nuclei of immortalized and malignant mouse lymphocytes. Cytom. Part A J. Int. Soc. Anal. Cytol..

[54] Bax B.E. (2022). Biomarkers in Rare Diseases 2.0. Int. J. Mol. Sci..

[55] Felman P., Bryon P., Gentilhomme O., Magaud J.P., Manel A.M., Coiffier B., Lenoir G. (1985). Burkitt’s lymphoma. Distinction of subgroups by morphometric analysis of the characteristics of 55 cell lines. Anal. Quant. Cytol. Histol..

[56] El Hussein S., Chen P., Medeiros L.J., Wistuba I.I., Jaffray D., Wu J., Khoury J.D. (2022). Artificial intelligence strategy integrating morphologic and architectural biomarkers provides robust diagnostic accuracy for disease progression in chronic lymphocytic leukemia. J. Pathol..

[57] Hayflick L., Moorhead P.S. (1961). The serial cultivation of human diploid cell strains. Exp. Cell Res..

[58] Bodnar A.G., Ouellette M., Frolkis M., Holt S.E., Chiu C.-P., Morin G.B., Harley C.B., Shay J.W., Lichtsteiner S., Wright W.E. (1998). Extension of life-span by introduction of telomerase into normal human cells. Science.

[59] Soudet J., Jolivet P., Teixeira M.T. (2014). Elucidation of the DNA end-replication problem in Saccharomyces cerevisiae. Mol. Cell.

[60] Blackburn E.H., Epel E.S., Lin J. (2015). Human telomere biology: A contributory and interactive factor in aging, disease risks, and protection. Science.

[61] Carlund O., Thörn E., Osterman P., Fors M., Dernstedt A., Forsell M.N.E., Erlanson M., Landfors M., Degerman S., Hultdin M. (2024). Semimethylation is a feature of diffuse large B-cell lymphoma, and subgroups with poor prognosis are characterized by global hypomethylation and short telomere length. Clin. Epigenetics.

[62] Montero-Conde C., Leandro-García L.J., Martínez-Montes Á.M., Martínez P., Moya F.J., Letón R., Gil E., Martínez-Puente N., Guadalix S., Currás-Freixes M. (2022). Comprehensive molecular analysis of immortalization hallmarks in thyroid cancer reveals new prognostic markers. Clin. Transl. Med..

[63] Hasenclever D., Diehl V., Armitage J.O., Assouline D., Björkholm M., Brusamolino E., Canellos G.P., Carde P., Crowther D., Cunningham D. (1998). A prognostic score for advanced Hodgkin’s disease. N. Engl. J. Med..

[64] Moccia A.A., Donaldson J., Chhanabhai M., Hoskins P.J., Klasa R.J., Savage K.J., Shenkier T.N., Slack G.W., Skinnider B., Gascoyne R.D. (2012). International Prognostic Score in advanced-stage Hodgkin’s lymphoma: Altered utility in the modern era. J. Clin. Oncol..

[65] Gautam S., Yeola S., Nahar A., Sarpong E.M., Prescott J., Yang X., Sineshaw H.M. (2023). Unfavorable early-stage Hodgkin lymphoma: Assessment of patient characteristics in a real-world setting. Future Oncol..

[66] Kumar A., Burger I.A., Zhang Z., Drill E.N., Migliacci J.C., Ng A., LaCasce A., Wall D., Witzig T.E., Ristow K. (2016). Definition of bulky disease in early stage Hodgkin lymphoma in computed tomography era: Prognostic significance of measurements in the coronal and transverse planes. Haematologica.

[67] Mohite A., Rangarajan V., Goda J., Chugh S., Agrawal A., Sengar M. (2023). Metabolic tumor parameters complement clinicopathological factors in prognosticating advanced stage Hodgkin Lymphoma. Asia Ocean. J. Nucl. Med. Biol..

[68] Wang Q., Qin Y., Zhou S., He X., Yang J., Kang S., Liu P., Yang S., Zhang C., Gui L. (2016). Prognostic value of pretreatment serum beta-2 microglobulin level in advanced classical Hodgkin lymphoma treated in the modern era. Oncotarget.

[69] Primerano S., Burnelli R., Carraro E., Pillon M., Elia C., Farruggia P., Sala A., Vinti L., Buffardi S., Basso G. (2016). Kinetics of circulating plasma cell-free dna in paediatric classical Hodgkin lymphoma. J. Cancer.

[70] Maco M., Kupcova K., Herman V., Kozak T., Mocikova H., Havranek O. (2022). Circulating tumor DNA in Hodgkin lymphoma. Ann. Hematol..

[71] Driessen J., Kersten M.J., Visser L., Berg A.v.D., Tonino S.H., Zijlstra J.M., Lugtenburg P.J., Morschhauser F., Hutchings M., Amorim S. (2022). Prognostic value of TARC and quantitative PET parameters in relapsed or refractory Hodgkin lymphoma patients treated with brentuximab vedotin and DHAP. Leukemia.

[72] Ansell S.M. (2022). Hodgkin lymphoma: 2023 update on diagnosis, risk-stratification, and management. Am. J. Hematol..

[73] Caputo V., Ciardiello F., Della Corte C.M., Martini G., Troiani T., Napolitano S. (2023). Diagnostic value of liquid biopsy in the era of precision medicine: 10 years of clinical evidence in cancer. Explor. Target. Anti-Tumor Ther..

